# Metal-insulator transition in a semiconductor nanocrystal network

**DOI:** 10.1126/sciadv.aaw1462

**Published:** 2019-08-23

**Authors:** Benjamin L. Greenberg, Zachary L. Robinson, Yilikal Ayino, Jacob T. Held, Timothy A. Peterson, K. Andre Mkhoyan, Vlad S. Pribiag, Eray S. Aydil, Uwe R. Kortshagen

**Affiliations:** 1 Department of Mechanical Engineering, University of Minnesota, Minneapolis, MN, USA.; 2 School of Physics and Astronomy, University of Minnesota, Minneapolis, MN, USA.; 3 Department of Chemical Engineering and Materials Science, University of Minnesota, Minneapolis, MN, USA.

## Abstract

Many envisioned applications of semiconductor nanocrystals (NCs), such as thermoelectric generators and transparent conductors, require metallic (nonactivated) charge transport across an NC network. Although encouraging signs of metallic or near-metallic transport have been reported, a thorough demonstration of nonzero conductivity, σ, in the 0 K limit has been elusive. Here, we examine the temperature dependence of σ of ZnO NC networks. Attaining both higher σ and lower temperature than in previous studies of ZnO NCs (*T* as low as 50 mK), we observe a clear transition from the variable-range hopping regime to the metallic regime. The critical point of the transition is distinctly marked by an unusual power law close to σ ∝ *T*^1/5^. We analyze the critical conductivity data within a quantum critical scaling framework and estimate the metal-insulator transition (MIT) criterion in terms of the free electron density, *n*, and interparticle contact radius, ρ.

## Teaser

Low-temperature conductivity measurements of ZnO nanocrystal networks illuminate a distinctive metal-insulator transition.

## INTRODUCTION

The metallic state is characterized by charge delocalization and the absence of an energy barrier to charge transport. Hence, a metal can be identified by measuring the temperature dependence of the conductivity, σ, and determining that σ(*T*) extrapolates to a nonzero value in the limit *T*→0. In most materials, the metal-insulator transition (MIT) is a continuous quantum phase transition (*1*, *2*)—driven by a physical tuning parameter, *p* (e.g., doping level)—near which the temperature dependence of σ can be described by a phenomenological scaling law of the form

$$\sigma =\sigma_{c}f(\Delta p/T^{y})$$ (1)

where *f* is a scaling function with two branches (insulating and metallic), Δ*p* denotes distance from the critical value of *p* (Δ*p* = |*p* – *p*_c_|), and σ_c_ ∝ *T^x^*, where *x* is typically ^1^/_2_ (*3*) or ^1^/_3_ (*4*) (typical values of *y* will be discussed later). For an MIT of this type, *p*_c_ can be determined by varying *p*, identifying the unique σ(*T*) curve that follows a power law, and then fitting all σ(*T*) data to Eq. 1.

For networks of semiconductor nanocrystals (NCs) joined at small facets of radius ρ, the MIT occurs at some critical value of *n*ρ^3^. The theoretical critical value (*5*, *6*) was found to be

$${\left(n\rho^{3}\right)}_{c}\approx 0.3g$$ (2)

where *g* is the degeneracy of conduction band minima. For ρ ≈ 1 nm, the MIT theoretically occurs near a critical density *n*_c_ ≈ 10^20^ to 10^21^ cm^−3^, which is ~2 to 3 orders of magnitude greater than a typical critical density predicted by the Mott criterion for bulk semiconductors (*5*). In previous experimental studies of charge transport in semiconductor NC networks, *n* has been increased by impurity doping (*5*) or by photodoping (*7*), and ρ has been increased by sintering (*7*) or by conformal deposition of additional semiconductor material onto connected NCs (*8*–*11*). Promising signs of metallic or near-metallic transport have emerged from studies of a variety of NC materials (*5*, *7*, *9*, *12*–*18*), including ZnO, for which *g* = 1. Lanigan and Thimsen (*9*) used atomic layer deposition (ALD) of ZnO onto ZnO NCs to attain conductivity, σ, greater than 30 ohm^−1^ cm^−1^ at room temperature and electron localization length, ξ, many times larger than the NC diameter, and subsequently, Greenberg *et al*. (*7*) obtained similar results from photonic sintering and photodoping. However, these reports lacked σ data below 2 K, and neither could thoroughly demonstrate σ(*T*→0) > 0.

In this work, we induce an MIT in ZnO NC networks by combining photonic sintering, ZnO ALD, and photodoping. We produce the NC networks via nonthermal plasma synthesis and supersonic impact deposition, using methods described previously (*7*, *19*). All networks receive the same photonic sintering and ALD treatments, and all have ρ = 2.8 ± 0.1 nm, *d* = 10.4 ± 0.2 nm, *t* = 300 ± 20 nm, and ϕ = 0.47 ± 0.02, where *d* is the average NC diameter based on x-ray diffraction (XRD) and ellipsometry, *t* is the film thickness, and ϕ is the ZnO volume fraction; ρ/*d* and *t*/ϕ ranges are based on sample-to-sample standard deviations (SDs) of XRD and ellipsometry measurements, respectively (see Materials and Methods for details on measurements and intrasample deviation). The tuning parameter in this study is the network’s free electron density, *n*, which we modulate by varying the degree of photodoping. We measure both the Hall effect (fig. S1) and localized surface plasmon resonance (LSPR) absorption, which yield *n*_Hall_ and *n*_LSPR_. We will show that these can be interpreted as measures of a network’s global average electron density and maximum local electron density, respectively.

## RESULTS AND DISCUSSION

**Fig. 1:**
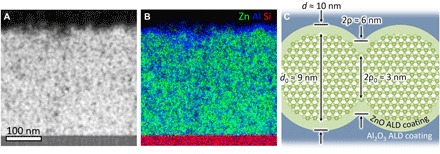
Anatomy of a metallic NC network. (**A**) Scanning transmission electron micrograph (STEM; high-angle annular dark-field image) of a metallic ZnO NC network lamella milled by focused ion beam. The lamella thickness is ~50 nm (about five NC diameters). (**B**) Corresponding composite energy-dispersive x-ray (EDX) spectral image showing counts of Zn (green), Al (blue), and Si (red). (**C**) Schematic showing approximate dimensions of a pair of neighboring NCs within the network with diameter, *d*, and interparticle contact radius, ρ (see text for exact values and intersample deviation). *d*_0_ and ρ_0_ are the values of an as-deposited NC network (before ZnO ALD), estimated from x-ray diffractometry (XRD) and ellipsometry data (*7*), respectively. *d* and ρ are estimated from the increase in ZnO volume fraction, ϕ, due to ZnO ALD (see Materials and Methods for details on measurement and intrasample deviation).

A micrograph, elemental map, and schematic of a fully treated ZnO NC network are shown in Fig. 1, and fabrication details are provided in Materials and Methods. After ρ is increased by ZnO ALD, the remaining pores are filled by Al_2_O_3_ ALD, which reduces the concentration of electron-trapping surface OH groups and enables high air-stable σ (*19*, *20*). Both ALD infills are conformal and spatially uniform (figs. S2 to S4), and the ZnO ALD infill is partially epitaxial (fig. S5). The NCs are not intentionally doped with impurities, and we assume that free electrons originate from oxygen vacancies, although we cannot rule out other donors such as hydrogen. Photodoping is accomplished by intense pulsed light (IPL) exposure after Al_2_O_3_ infilling, which leads to persistent enhancement of σ (*7*, *21*). Previous studies of photoconductive ZnO (*7*, *21*, *22*) have linked this effect to photooxidation of electron-trapping adsorbates (residual OH in the case of Al_2_O_3_-infilled ZnO NC networks; see note S1) that eliminates surface states and transfers electrons to the conduction band.

To produce the main MIT dataset, we vary the number of photodoping IPL flashes from 100 to 1000 so that *n*_Hall_ ranges from 2.2 × 10^19^ to 6.9 × 10^19^ cm^−3^ and (*n*ρ^3^)_Hall_ ranges from 0.49 to 1.5 (see Materials and Methods for details on Hall measurements and associated uncertainty in *n* and *n*ρ^3^). This *n* range is well above the critical *n* for the Mott MIT, *n* ≈ 4 × 10^18^ cm^−3^ (*7*), and therefore, the individual NCs are expected to behave metallically in all samples. At 300 K, the corresponding electron mobility ranges from 4.5 to 8.3 cm^2^ V^−1^ s^−1^ and σ ranges from 16 to 92 ohm^−1^ cm^−1^, an upper bound approximately three times higher than that attained in previous studies of ZnO NCs (*7*, *9*). Translating σ to interparticle conductance, *G*, via the three-dimensional (3D) nodes and links model (*23*) [*G* = σ*d*(σ – σ_0_)^1.9^, σ_0_ ≈ 0.2 (*24*)], we obtain a *G* range of ~2 × 10^−4^ to 10 × 10^−4^ ohm^−1^, which lies above the quantum conductance, e^2^/πħ ≈ 8 × 10^−5^ ohm^−1^.

**Fig. 2:**
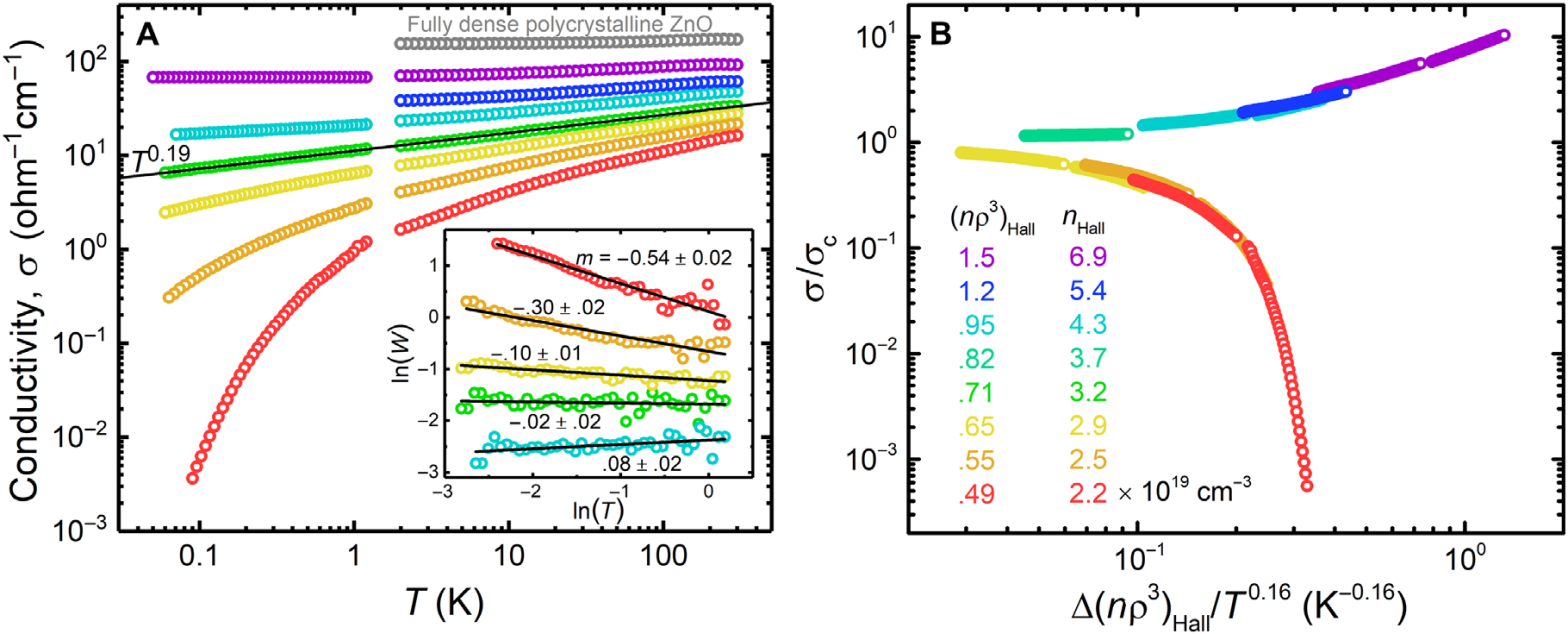
Conductivity, σ(*n*ρ^3^, *T*), across the MIT. (**A**) Log-log plot of σ versus *T*. The colored curves correspond to ZnO NC networks with *n*ρ^3^ varied by photodoping, whereas the gray curve was obtained from a fully dense polycrystalline ZnO film grown by ALD. The black line is a linear fit of logσ versus log*T* at (*n*ρ^3^)_Hall_ = 0.71 (green curve), which indicates σ ∝ *T*^0.19^. Inset: ln*W* versus ln*T* at low *T*, where *W* = d(lnσ)/d(ln*T*), so that the sign of slope, *m*, corresponds to the sign of curvature in the main plot. The curve for (*n*ρ^3^)_Hall_ = 0.82 is omitted to aid visualization of the linearity of the critical curve. (**B**) Collapse of the insulating and metallic σ(*T*) data from 50 mK to 180 K into the two branches of the scaling function in Eq. 1, with *p* = (*n*ρ^3^)_Hall_, (*n*ρ^3^)_c_ = 0.71, and *y* = 0.16.

The evolution of σ versus *T* between 300 K and 50 mK, shown in Fig. 2A, provides clear evidence of an MIT. For (*n*ρ^3^)_Hall_ < 0.71, σ has a strong *T* dependence: σ(*T*) has positive slope and negative curvature on a log-log scale, indicating that σ vanishes in the limit *T*→0. In contrast, for (*n*ρ^3^)_Hall_ > 0.71, σ(*T*) has positive curvature, which strongly suggests σ(*T*→0) > 0. Indeed, between 1.2 K and 50 mK, the change in σ of the most conductive NC network [(*n*ρ^3^)_Hall_ = 1.5, purple curve] is within experimental error (<1%). Furthermore, this sample has negative dσ/d*T* at high *T* (shown more clearly in fig. S6), suggesting a crossover to the phonon scattering regime expected in metals. However, negative dσ/d*T* is not exhibited exclusively by metals (*25*–*27*), and we emphasize that our key finding is evidence of nonzero σ in the 0 K limit.

At (*n*ρ^3^)_Hall_ = 0.71 (green curve), between the insulating and metallic regimes, σ follows a power law close to σ ∝ *T*^1/5^. As shown in Fig. 2A, a linear fit of logσ versus log*T* yields an exponent of 0.19. Power-law behavior at (*n*ρ^3^)_Hall_ = 0.71 is confirmed by a Zabrodskii plot of ln*W* versus ln*T* at low *T* (inset of Fig. 2A), where *W* = d(lnσ)/d(ln*T*). The evolution of slope on this plot from negative to zero to positive reflects the transition from negative to positive curvature in the main plot, with a power law occurring uniquely at the dividing line, namely, the MIT critical point. [In addition, the appearance on the Zabrodskii plot of a straight line with slope near −^1^/_2_ reveals that the transport mechanism at low *n*ρ^3^ is Efros-Shklovskii variable-range hopping (*28*), in agreement with previous studies of similar ZnO NCs (*7*, *9*).]

As shown in Fig. 2B, the σ versus *T* data can be described by the critical scaling law, Eq. 1, with *p* = (*n*ρ^3^)_Hall,_ (*n*ρ^3^)_c_ = 0.71, and *y* = 0.16: All data collapse into two branches corresponding to the insulating and metallic sides of the MIT. (By testing a range of values, we have determined that *y* = 0.16 ± 0.02.) With (*n*ρ^3^)_c_ = 0.71, the distance from the critical point, Δ(*n*ρ^3^), ranges from 0.2 to 0.8, and Δ(*n*ρ^3^)/(*n*ρ^3^)_c_ ranges from 0.3 to 1.1. Assuming an effective mass of 0.3*m*_e_, the 3D free-electron Fermi temperature ranges from ~1000 to 2000 K, which allows for applying scaling analysis at relatively high temperature (*29*). We use a range of 50 mK to 180 K, above which phonon scattering becomes the dominant influence on σ(*T*) for metallic samples (for example, see fig. S6). According to critical scaling theory (*2*, *3*), *y* = 1/*vz*, where *v* and *z* are the critical exponents of the diverging length and time scales, i.e., ξ ∝ [Δ(*n*ρ^3^)]^−*v*^ and τ ∝ ξ*^z^*. The temperature exponent at the critical point, *x* = 0.19, is equal to μ/*vz*, where μ is the conductivity exponent, i.e., σ(*T*→0) ∝ [Δ(*n*ρ^3^)]^μ^. Typically, μ ≈ 1. Our finding that *x* = 0.19 and *y* = 0.16 ± 0.02 suggests that μ = 1.2 ± 0.2. More remarkably, *y* = 0.16 ± 0.2 translates to *vz* = 6.3 ± 0.8, whereas in most MITs, *vz* ≤ 3 (*2*–*4*). Results of a previous study of near-MIT transport in ZnO NCs appeared consistent with *vz* = 2 (*7*), although the present data suggest that this was due to underestimated distance from the MIT (see note S2). High *vz* has been observed previously in materials considerably different from ours, namely, the highly correlated Mott-Hubbard systems NiS_2−*x*_Se*_x_* and YH*_x_*, which exhibited *vz* ≈ 5 (*30*) and *vz* ≈ 6 (*31*), respectively. Currently, we are unable to identify the cause of high *vz* in ZnO NC networks, although we note that the critical frequency exponent of σ—and hence the critical temperature exponent, μ/*vz*—depends on dimensionality (*32*, *33*). A question to consider is whether conduction in a porous NC network can have an associated fractal dimension less than three that gives rise to low μ/*vz* (see note S3). However, our finding that *vz* = 6.3 ± 0.8 should be interpreted cautiously until additional low-*T* data are obtained on the metallic side of the transition. In general, *vz* can be cross-checked by directly determining μ from a fit of σ(*T*→0) extrapolations plotted against the tuning parameter [in our case, Δ(*n*ρ^3^)] on the metallic side (*2*). This analysis would require additional metallic samples and possibly measurements at temperatures below the current range (*3*, *34*).

**Fig. 3:**
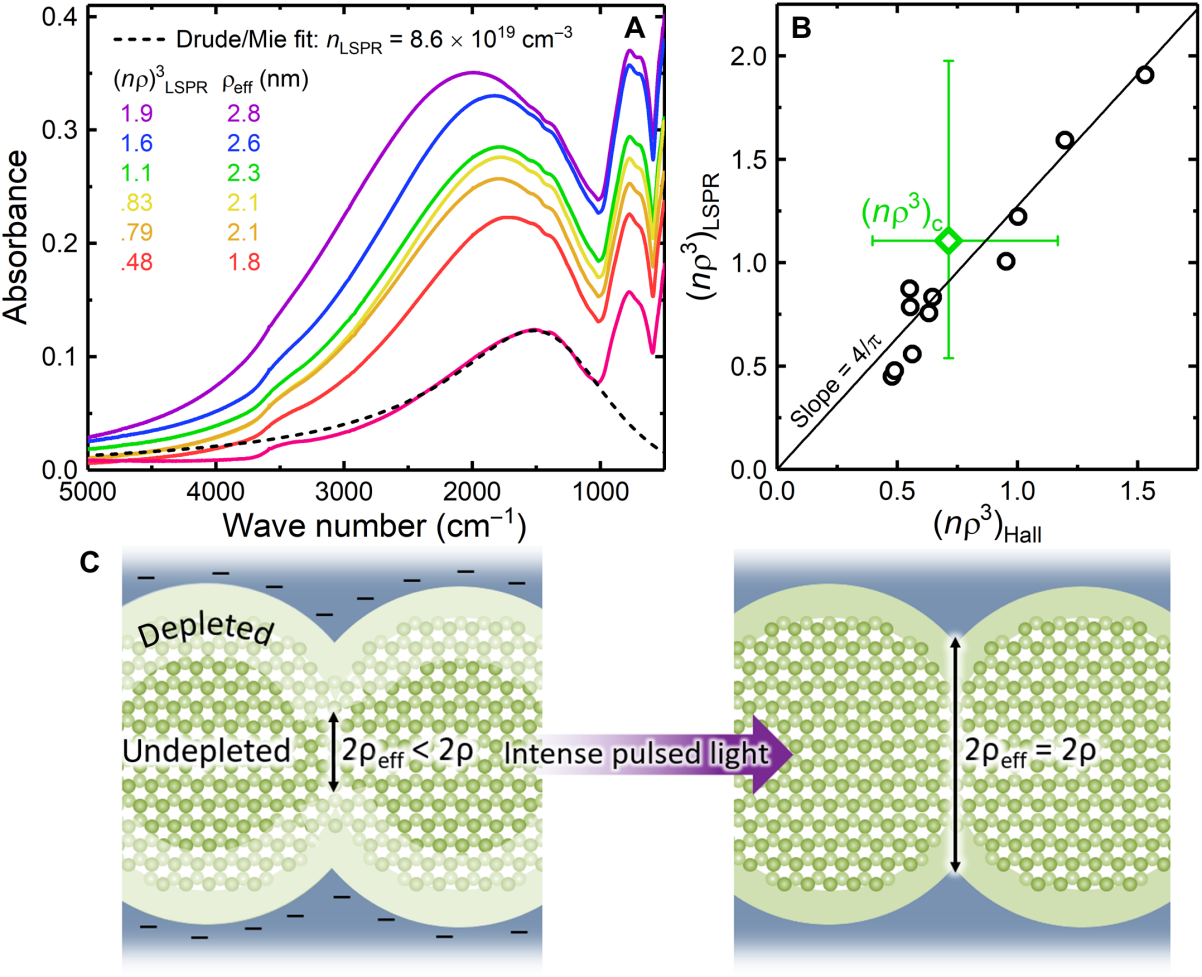
Estimating *n*ρ^3^ from LSPR absorption. (**A**) Select IR absorption spectra showing LSPR absorption. All spectra correspond to σ(*T*) data in Fig. 2, except for that showing the weakest absorption (magenta), which was acquired from a sample farther from the MIT. (**B**) Comparison of two estimates of *n*ρ^3^. (*n*ρ^3^)_LPSR_ = *n*_LSPR_ × ρ_eff_^3^, whereas (*n*ρ^3^)_Hall_ is the product of *n*_Hall_ and the ρ^3^ defined by the ZnO lattice, shown in Fig. 1C. The error bars on the (*n*ρ^3^)_c_ point represent an uncertainty in ρ of ±0.5 nm. All acquired IR spectra are represented in (B), while outlying spectra are excluded from (A) to clarify the trend. (**C**) Surface depletion model.

Having established that the MIT apparently occurs at (*n*ρ^3^)_Hall_ = 0.71, we now obtain another estimate of the MIT criterion by analyzing the NCs’ LSPR absorption and the mechanism by which *n*ρ^3^ increases. As shown in Fig. 3A, the evolution of the LSPR absorption feature is unexpected: The feature intensifies with increasing σ, but the corresponding blueshift of the absorption peak frequency, ω_peak_, is smaller than expected from conventional absorption models (*35*, *36*). Previously, it was suggested that such behavior could be caused by divergence of the dielectric constant near the MIT (*7*), but we have ruled out this explanation by measuring ultraviolet (UV)–induced LSPR evolution far from the MIT and observing similar blueshift suppression (fig. S7). Instead, we attribute this LSPR behavior to depletion layers at the NC surfaces. In a recent study of In_2_O_3_:Sn NCs by Zandi *et al*. (*37*), which built upon the work of zum Felde *et al*. (*38*), blueshift suppression was connected to depletion due to band bending (Fermi level pinning) by surface states. The authors charged the NCs electrochemically to counteract the band bending and found that in sufficiently large and/or heavily doped NCs, ω_peak_ remained nearly constant as absorbance increased, which they interpreted as an enhancement of the undepleted core volume (reduction of surface depletion width) such that the total number of free electrons increased, while the free electron density in the undepleted cores remained nearly constant. As depicted in Fig. 3C, we propose that our system behaves similarly when photodoped by IPL: The ZnO NCs have depleted surfaces due to filled surface states, and IPL reduces the depletion width by eliminating these states and promoting the electrons to the NC conduction bands. For additional experimental evidence in support of this model, see the recent report by Benton *et al*. (*39*) on hopping conduction in similar ZnO NC networks, which exhibited constant density of states at the Fermi level as electron localization length was increased by UV irradiation.

Figure 3B compares the two estimates of *n*ρ^3^. Whereas we obtain (*n*ρ^3^)_Hall_ by assuming constant ρ and variable *n* (*n*_Hall_), we obtain (*n*ρ^3^)_LSPR_ by assuming constant *n* and variable ρ. That is, we approximate the electron density within undepleted regions as a constant determined by the presumably spatially uniform donor density (we neglect the small LSPR blueshift), and we take ρ to be not the fixed contact radius determined by the ZnO lattice but rather a smaller effective contact radius between undepleted regions, ρ_eff_ (see Fig. 3C). To estimate *n*_LSPR_ without delving into the evolution of interparticle coupling (*40*, *41*), we use Mie theory to fit the LSPR absorption feature of a sample far from the MIT (magenta spectrum in Fig. 3A), which we approximate as an ensemble of isolated cores of conductive ZnO embedded in depleted ZnO. We then estimate ρ_eff_ by assuming that the undepleted volume is proportional to the area under the LSPR absorption feature (*42*), and that ρ_eff_ approaches ρ as depletion width approaches zero (see Materials and Methods for details).

As seen in Fig. 3B, the resultant (*n*ρ^3^)_LSPR_ are only slightly larger than the corresponding (*n*ρ^3^)_Hall_, and good agreement can be obtained by applying a correction factor of 4/π, which is the expected ratio of *n* within an NC to *n*_Hall_ for cubically packed NCs at high temperature and on the metallic side of the MIT (*43*). This agreement lends confidence to our independent measurements of *n* and suggests that error in ρ is what limits the accuracy of our estimate of (*n*ρ^3^)_c_. Although the sample-to-sample SD of ρ is small (± 0.1 nm), which makes scaling analysis possible, systematic error in ρ may be high due to the simplifying assumptions of our geometric model. Assuming an uncertainty of ±0.5 nm (comparable to the ZnO ALD coating thickness), (*n*ρ^3^)_c_ ranges from ~0.5 to 2 (error bars in Fig. 3B). This is consistent with the proposition—suggested by the results of (*7*, *9*)—that Eq. 2 errs toward underestimating the MIT criterion for at least these particular [porous and disordered (*7*)] NC networks. In future studies of Eq. 2, this hypothesis should be tested by relatively direct measurements of ρ, perhaps by electron tomography.

In this work, we have confirmed that an MIT is achievable in a semiconductor NC network by manipulation of *n* and ρ. Our σ versus *T* data suggest that in our ZnO NC networks, the MIT is a continuous quantum phase transition with an unusual power law close to σ ∝ *T*^1/5^ at the critical point. We have estimated *n*ρ^3^ in the critical region from measurements of both the Hall effect and the LSPR, which we have reconciled with a simple NC surface depletion model. Our observation of clear metallic behavior and σ merely two times lower than that of a fully dense polycrystalline ZnO film (Fig. 2A) bodes well for the development of NC materials that combine bulk-like charge transport with advantages of nanostructuring. For example, the transparency and nanoporosity of these ZnO NC networks could be exploited (the Al_2_O_3_ infill could be replaced with other materials) to create conductive nanocomposites with high interface area and size-tunable optical properties.

## MATERIALS AND METHODS

### NC network fabrication and treatment

The plasma synthesis, IPL, and ALD methods have been described previously (*7*, *19*). ZnO NCs were synthesized in a radio-frequency plasma and deposited by supersonic impact onto borosilicate substrates [or NaCl substrates for Fourier transform infrared (FTIR) measurements] to form networks with *d*_0_ ≈ 9 nm and ϕ_0_ ≈ 0.33. The as-deposited NC surfaces are terminated primarily in hydroxyl and carboxylate groups. Each network was sintered by 1000 flashes of IPL applied at a surface power density of 12 kW/cm^2^ with a 1-ms pulse length and 660-ms period, using a Xenon Corp. Sinteron 2010 equipped with a 10-inch Xe U-lamp. As shown previously (*7*), these conditions give ρ_0_ ≈ 1.5 nm. Subsequently, to increase ρ further, we conformally coated the NCs with additional ZnO by eight cycles of ALD, using a Cambridge Nanotech/Ultratech Savannah S200 system. ZnO ALD was followed by 70 cycles of Al_2_O_3_ ALD. The ALD precursors were diethylzinc, trimethylaluminum, and water. The precursor pulse times were 100 ms, the purge time between pulses was 30 s, and the deposition temperature was 180°C. After ALD, the NCs were photodoped by additional IPL flashes, using settings identical to those of the sintering treatment. XRD patterns were unchanged by sintering and photodoping IPL, indicating that neither treatment increased the average ZnO crystallite size.

The NCs were exposed to air immediately after synthesis/deposition, and IPL was applied under ambient conditions. In dark ambient conditions, σ of a fully treated sample decays at a rate of up to ~1% per day. Between measurements, the samples were stored in dark inert conditions (under N_2_) so that σ was stable for at least several months. Some low-*T* (<2 K) measurements were performed months after the corresponding high-*T* measurements of the same films, and the resultant mismatch at ~2 K was minimal (see Fig. 2A).

To gauge synthesis and treatment repeatability, some samples were produced twice under nominally identical conditions. For a given set of conditions, σ(300 K), (*n*ρ^3^)_Hall_, and (*n*ρ^3^)_LSPR_ all varied by up to ~50%. A number of experimental factors may contribute to this variation, including exposure to ambient light and humidity and slight variability in sample position under the IPL lamp. To prevent impact on scaling analysis, we ensured that each σ measurement was paired with *n*ρ^3^ measurements of the same sample—or effectively the same for (*n*ρ^3^)_LSPR_ measurements, for which NaCl-substrate samples were produced and treated simultaneously alongside the corresponding borosilicate-substrate samples. In total, ~20 samples were produced to obtain a range of σ, and nine samples were selected for low-temperature measurements and scaling analysis based on the spacing of their σ(300 K). Of these nine samples, eight were found to follow a near-linear relationship between σ(300 K) and *n*_Hall_ indicative of consistent film structure and thickness, while one was found to have outlying (high) σ(300 K) and was therefore excluded.

The fully dense polycrystalline ZnO reference film (gray curve in Fig. 2A) was deposited at 180°C using an expedited ZnO ALD recipe: The diethylzinc and water pulse times were 15 ms, and the purge time between pulses was 5 s. ALD was carried out for 1000 cycles to produce a film approximately 170 nm in thickness.

### Structural characterization

XRD patterns were acquired using a Bruker D8 Discover diffractometer equipped with a Be area detector and a Co Kα source. Average crystallite size was determined by Scherrer analysis of the (100), (002), and (101) peaks (see fig. S5). As-deposited NC diameter, *d*_0_, was assumed to be equal to this crystallite size; previously, this assumption was corroborated by electron micrographs. NC film thickness and porosity were measured before Al_2_O_3_ deposition with a J.A. Woollam M44 ellipsometer. The backsides of the borosilicate substrates were roughened with a grinding wheel to prevent reflection. Film thickness, *t*, and ZnO volume fraction, ϕ, were determined by fitting ellipsometry spectra in WVASE, assuming two components (ZnO and void) and using the Bruggeman effective medium approximation.

We estimate fully treated NC diameter, *d*, and interparticle contact radius, ρ, from the increase in ϕ due to ZnO ALD (from ϕ_0_ ≈ 0.33 to ϕ ≈ 0.47). We approximate the ALD-coated NCs as spheres enveloped in partial spherical shells with thickness δ ≈ *d*_0_[(ϕ/ϕ_0_)^1/3^ – 1]/2 so that ρ is given by (ρ_0_^2^ + δ^2^ + δ*d*_0_)^1/2^ and *d* is given by *d*_0_ + 2δ. Note that this method yields δ ≈ 0.6 nm, whereas from the ZnO growth per cycle (GPC) on a smooth substrate under the same ALD conditions (0.16 nm), one might predict δ = 8 × 0.16 nm = 1.3 nm. Evidently, GPC is lower within a ZnO NC network.

Previous ellipsometry measurements at multiple locations of ZnO NC networks have shown that the intrasample SDs of *t*_0_ and ϕ_0_ are 30 nm and 0.02, respectively (about the same as the sample-to-sample deviations). Previous transmission electron microscopy (TEM) images [e.g., those shown in (*44*)] have revealed that the intrasample SD of *d*_0_ (NC size dispersity) is ~2 nm. Assuming that ρ_0_ is approximately proportional to *d*_0_, this translates to a ρ_0_ dispersity of ~0.3 nm. Given that dρ/dδ does not depend strongly on ρ_0_, the dispersity in ρ is likewise ~0.3 nm.

### Electron microscopy

To characterize the structure and ALD infill uniformity of the NC networks, we prepared TEM cross-sectional lamellae using an FEI Helios NanoLab G4 dual-beam focused ion beam and subsequently studied them using scanning TEM (STEM) and energy-dispersive x-ray (EDX) spectroscopy. An aberration-corrected FEI Titan G2 60-300 STEM equipped with a Super-X EDX spectrometer was operated at 60 kV with a convergence semiangle of 25 mrad and a beam current of 120 pA. Spatially resolved STEM-EDX maps were collected with 1024 pixels by 1024 pixels over 450 nm by 450 nm areas, a dwell time of 4 μs/pixel, an acquisition time of 10 min, and drift correction after every frame. The Zn, Al, O, Si, and C K-edges were background-subtracted and integrated, producing spectral images. A three-pixel Gaussian blur was applied to the final spectral images to reduce noise and aid in visualization.

### Electron transport measurements

Aluminum contact pads were deposited by thermal evaporation onto the corners of ZnO NC networks on 5 mm by 5 mm borosilicate substrates. In addition, because the backs and sides of the substrates were inevitably coated with conductive ZnO during the ZnO ALD step, the samples were mounted onto sapphire wafers with a thermally conductive varnish to prevent shorting through metallic sample holders during measurement.

Temperature-dependent conductivity measurements from 300 to 2 K were performed in the van der Pauw configuration with Quantum Design Physical Properties Measurement System (PPMS) with external electronics. Current through a pair of adjacent contacts was swept from −1 to 1 μA using a Keithley 220 current source, and the voltage between the other two contacts was measured with a Keithley 2182 nanovoltmeter. A Keithley 2700 switch box was used to perform the four-point measurements in both configurations, and ohmic behavior in four- and two-point configurations was confirmed at 300 and 2 K. Reported σ values were measured while *T* was incremented from 2 to 300 K and held at discrete values during the *IV* sweeps. Each resultant σ versus *T* curve was compared to the corresponding σ versus *T* data acquired during cooldown to confirm that drift in σ during measurement was ~1% or less. Data below 2 K were acquired using an Oxford Triton 200 dilution refrigerator. *T* was held at discrete values for 1 to 2 min to ensure stabilization. Conductivity measurements were performed in either DC or quasi-DC mode (13-Hz AC with a time constant of 1 s), using a custom IVVI rack with a high-precision current source, a Keithley 2000 voltmeter, and a Stanford Research Systems SR830 lock-in amplifier. *IV* scans were performed at the lowest *T*, and excitation currents in the linear *IV* regime were selected for *T* scans. Comparison of DC and quasi-DC measurements at the lowest *T* confirmed that the two excitation modes yield the same σ.

Hall measurements were performed with the PPMS at 300 K and a DC excitation of 10 μA. A film thickness of 300 nm was used for all Hall calculations. Uncertainty in *n*_Hall_ is due predominantly to uncertainty in film thickness; as stated above, inter- and intrasample SDs are ±20 and ±30 nm, respectively. Therefore, uncertainty in *n*_Hall_ is approximately ±10%.

### IR spectroscopy and LSPR modeling

FTIR spectra were collected under an N_2_ atmosphere from witness samples deposited on polished NaCl substrates, using a Bruker ALPHA spectrometer in transmission mode. We assume that all extinction is due to absorption. To estimate *n*_LSPR_, we used the Drude model and Mie theory to fit the LSPR absorption feature of the magenta spectrum in Fig. 3A. Our fitting procedure is as described in (*19*), except that we use ε = 3.7 as both the high-frequency dielectric constant of the conductive spheres (undepleted ZnO) and the dielectric constant of the surrounding medium (assumed to be depleted ZnO); the fitting error is approximately ±0.1 × 10^19^ cm^−3^. Then, to estimate ρ_eff_, we estimated the relative change in area under the LSPR feature. To avoid the error that would be introduced by fitting the Al_2_O_3_ feature at ~800 cm^−3^, we exploited the nearly constant LSPR lineshape and integrated the high-frequency half of each LSPR feature, i.e., the integration bounds are 6000 cm^−1^ and ω_peak_. The experimental data were integrated directly (trapezoidally) without peak fitting. We translated area to ρ_eff_^3^ by making the following assumptions:

1) The NC network has a continuous 3D depletion layer at the ZnO/Al_2_O_3_ interface, which extends into the ZnO to a width, *w*, that is uniform throughout the network. Therefore ρ_eff_ = ρ – *w*, and the largest cross section of the undepleted region has radius *r*_eff_ = *r* – *w*.

2) In our most conductive sample, we have *w* = 0 so that ρ_eff_ = ρ = 2.8 nm and *r*_eff_ = *r* = 5.2 nm. This assumption is supported, though not confirmed, by the finding that σ cannot be further increased by IPL.

3) The total undepleted volume, *V*_eff_, is proportional to *r*_eff_^3^.

4) Area under the LSPR absorption feature is proportional to the total number of free electrons [see (*42*)], which is proportional to the total undepleted volume.

For example, if a sample has an LSPR area half that of the most conductive sample, then we have *r*_eff_ = (0.5)^1/3^ × 5.2 nm = 4.1 nm, which implies *w* = (5.2 − 4.1) nm = 1.1 nm and therefore ρ_eff_ = (2.8 − 1.1) nm = 1.7 nm.

## Supplementary Material

http://advances.sciencemag.org/cgi/content/full/5/8/eaaw1462/DC1

Download PDF

## SUPPLEMENTARY MATERIALS

Supplementary material for this article is available at http://advances.sciencemag.org/cgi/content/full/5/8/eaaw1462/DC1 Note S1. Comment on correlation between LSPR and OH FTIR signals. Note S2. Comment on vz in previous ZnO NC networks. Note S3. Comment on the spectral fractal dimension. Fig. S1. Representative Hall data. Fig. S2. STEM/EDX images and depth profiles. Fig. S3. Composite STEM-EDX images. Fig. S4. Higher-magnification STEM/EDX images of the metallic network. Fig. S5. XRD before and after ZnO ALD. Fig. S6. Rescaled plot of σ(*T*) at (*n*ρ^3^)_Hall_ = 1.5. Fig. S7. UV enhancement of LSPR absorption far from the MIT.
