# Supplementary material for: Metal-insulator transition in a semiconductor nanocrystal network
Source: Sci Adv. 2019 Aug 23;5(8):eaaw1462. doi: 10.1126/sciadv.aaw1462 (PMC6707780; doi:10.1126/sciadv.aaw1462)
Supplement: http://advances.sciencemag.org/cgi/content/full/5/8/eaaw1462/DC1 [file supp_5_8_eaaw1462__index.html]

Science Advances | Science AdvancesAAASSearchScience AdvancesMenu

## Supplementary Materials

**This PDF file includes:**

- Note S1. Comment on correlation between LSPR and OH FTIR signals.
- Note S2. Comment on *vz* in previous ZnO NC networks.
- Note S3. Comment on the spectral fractal dimension.
- Fig. S1. Representative Hall data.
- Fig. S2. STEM/EDX images and depth profiles.
- Fig. S3. Composite STEM-EDX images.
- Fig. S4. Higher-magnification STEM/EDX images of the metallic network.
- Fig. S5. XRD before and after ZnO ALD.
- Fig. S6. Rescaled plot of σ(*T*) at (*n*ρ3)Hall = 1.5.
- Fig. S7. UV enhancement of LSPR absorption far from the MIT.

Download PDF

**Files in this Data Supplement:**

- Adobe PDF - aaw1462\_SM.pdf
