# Supplementary material for: Metal-insulator transition in a semiconductor nanocrystal network
Source: Sci Adv. 2019 Aug 23;5(8):eaaw1462. doi: 10.1126/sciadv.aaw1462 (PMC6707780; doi:10.1126/sciadv.aaw1462)
Supplement: Download PDF [file aaw1462_SM.pdf]

Science Advances
AAAS

advances.sciencemag.org/cgi/content/full/5/8/eaaw1462/DC1

Supplementary Materials for

Metal-insulator transition in a semiconductor nanocrystal network

Benjamin L. Greenberg*, Zachary L. Robinson, Yilikal Ayino, Jacob T. Held, Timothy A. Peterson, K. Andre Mkhoyan, Vlad S. Pribiag, Eray S. Aydil, Uwe R. Kortshagen*

*Corresponding author. Email: bengreenberg@umn.edu (B.L.G.); kortshagen@umn.edu (U.R.K.)

Published 23 August 2019, *Sci. Adv.* **5**, eaaw1462 (2019)
DOI: 10.1126/sciadv.aaw1462

This PDF file includes:

Note S1. Comment on correlation between LSPR and OH FTIR signals.
Note S2. Comment on *vz* in previous ZnO NC networks.
Note S3. Comment on the spectral fractal dimension.
Fig. S1. Representative Hall data.
Fig. S2. STEM/EDX images and depth profiles.
Fig. S3. Composite STEM-EDX images.
Fig. S4. Higher-magnification STEM/EDX images of the metallic network.
Fig. S5. XRD before and after ZnO ALD.
Fig. S6. Rescaled plot of $\sigma(T)$ at $(n\rho^3)_{Hall} = 1.5$.
Fig. S7. UV enhancement of LSPR absorption far from the MIT.

Notes:

S1. Comment on correlation between LSPR and OH FTIR signals. In (*7*), and in Fig. S7, LSPR intensification is strongly correlated with diminution of the OH absorption feature near 3500 cm^{-1}, which has been interpreted as removal of OH from the NC network upon photooxidation. In Fig. 3a, however, the correlation between LSPR and OH absorption is weak, suggesting that OH remains in the network after photooxidation, or that photooxidation of a species other than OH contributes to σ enhancement. In either case the main conclusions of this article are unaffected.

S2. Comment on *νz* in previous ZnO NC networks. Because the MIT was not crossed in (*7*), we cannot rule out the possibility that *νz* was in fact lower. The NC networks in that study were not identical to those in this work: they lacked a ZnO ALD coating. However, this lack of coating primarily amounts to lower ϕ, which is not expected to correspond to lower *νz*; see (*32*, *33*) and Note 3 *infra*.

S3. Comment on the spectral fractal dimension. Presumably this would be the spectral fractal dimension, $\tilde{d}$, also known as the fracton dimension. However, for most percolating networks, $\tilde{d} \approx 4/3$, and it has been shown that delocalization is impossible for $\tilde{d} < 2$; see (*33*). Thus the key question is whether our NC networks have an unusual $\tilde{d}$ between 2 and 3.

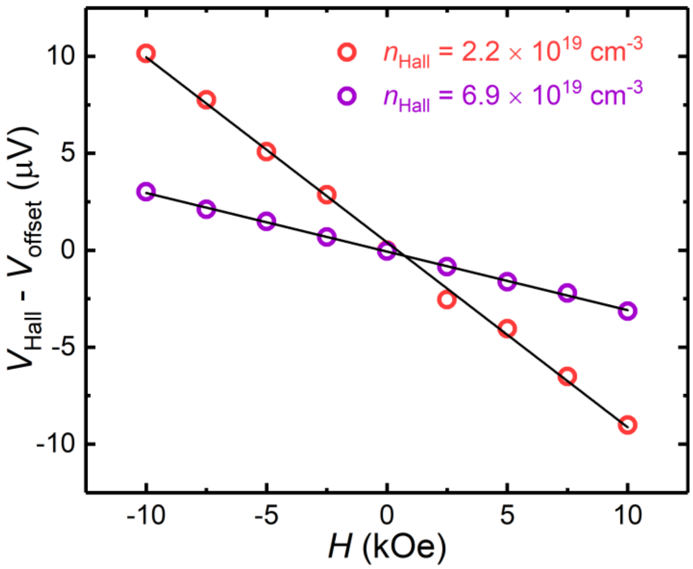

Fig. S1. Representative Hall data. Hall data corresponding to the least and most conductive samples. Offset voltages due to slight anisotropy of the samples are subtracted to zero the vertical intercepts.

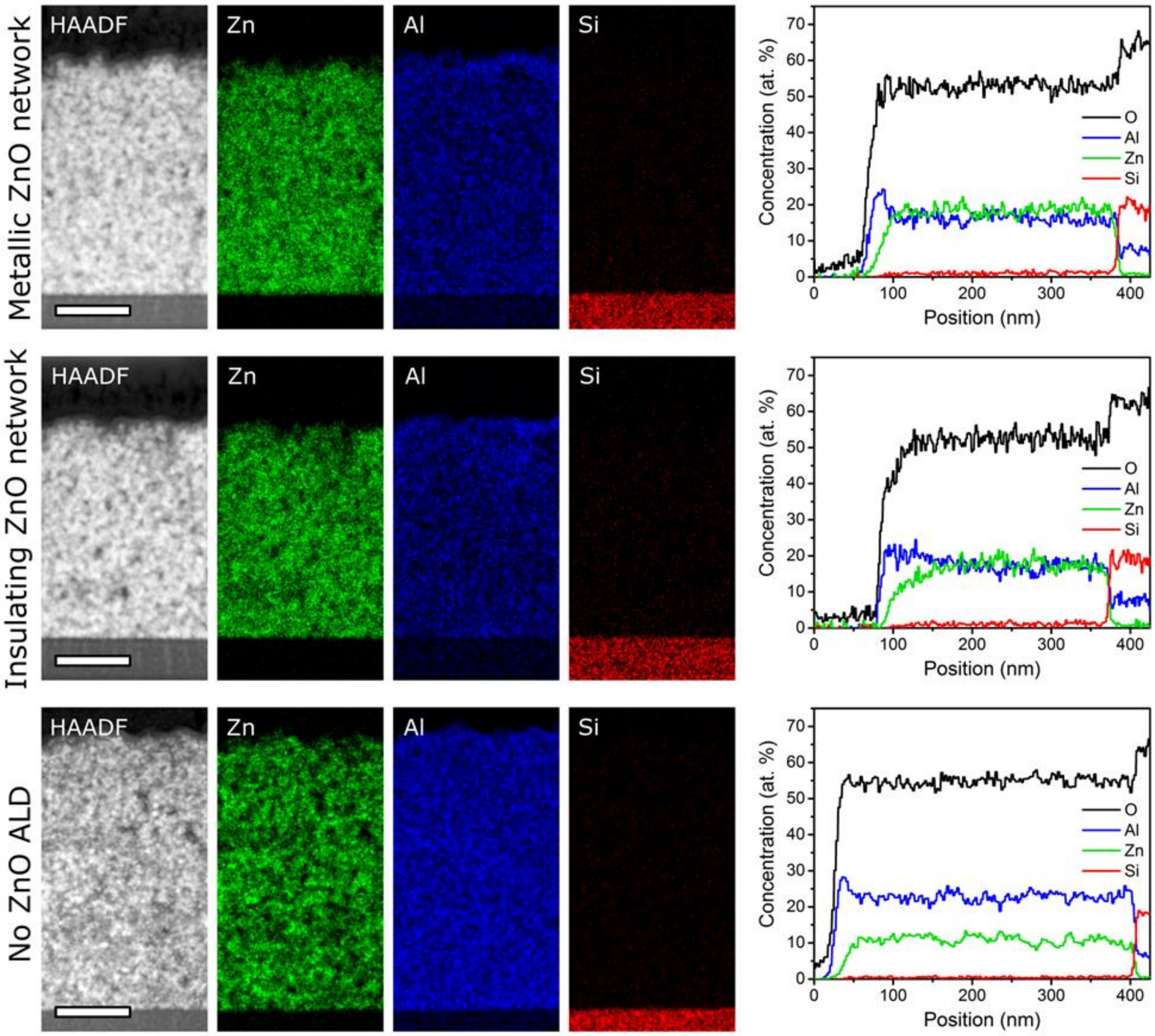

Fig. S2. STEM/EDX images and depth profiles. elemental distribution in ALD-infilled NC networks. The metallic ($(n\rho^3)_{\mathrm{Hall}} = 1.5$) and insulating ($(n\rho^3)_{\mathrm{Hall}} < 0.5$) networks are infilled with 8 cycles of ZnO ALD followed by 70 cycles of Al_2O_3 ALD, whereas the "No ZnO ALD" network is infilled with 70 cycles of Al_2O_3 ALD only. (Column 1) HAADF-STEM and EDX spectral images showing the raw counts for Zn, Al, and Si for each of the networks. (Column 2) Approximate atomic % of all relevant elements across the same regions, demonstrating uniform and conformal coating with both ZnO and Al_2O_3 throughout the films. All scale bars are 100 nm. Note that the approximate atomic % of Zn and Al are not equivalent to the volume fractions of ZnO and Al_2O_3 (which in the main text have been inferred from ellipsometry), and a rigorous comparison of these quantities is impractical without a measurement of the Al_2O_3 mass density.

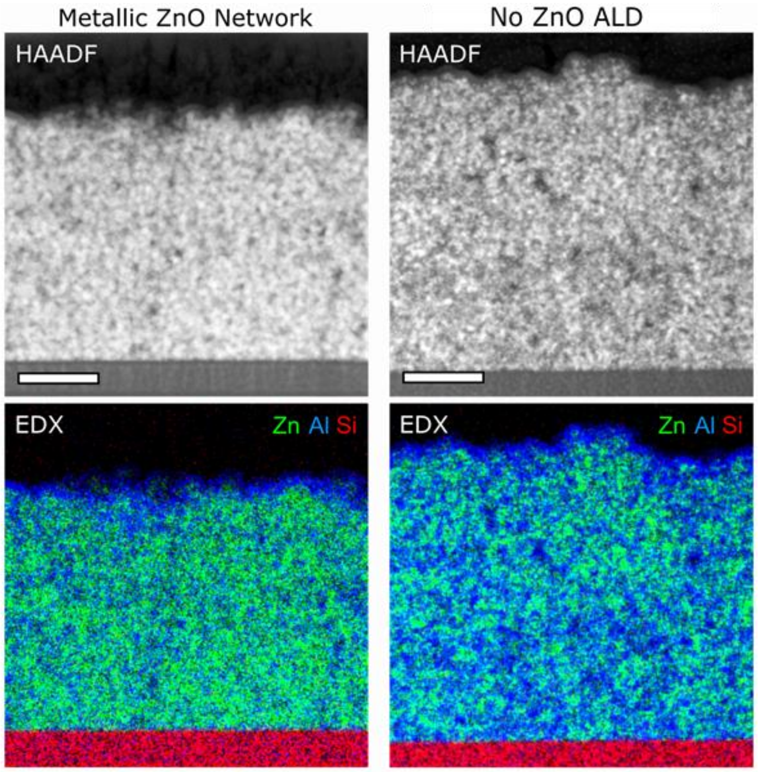

Fig. S3. Composite STEM-EDX images. Scale bars are 100 nm.

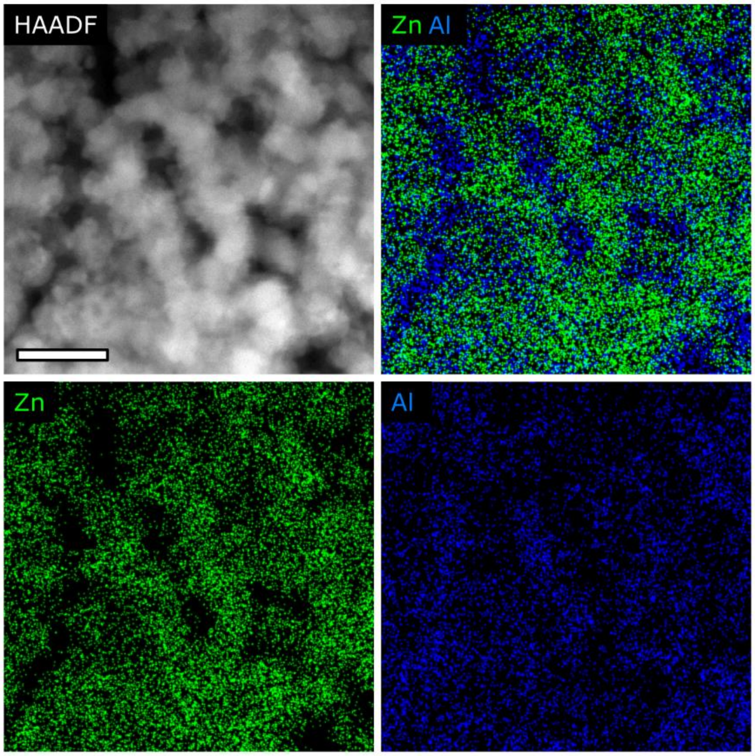

Fig. S4. Higher-magnification STEM/EDX images of the metallic network. Scale bar is 20 nm.

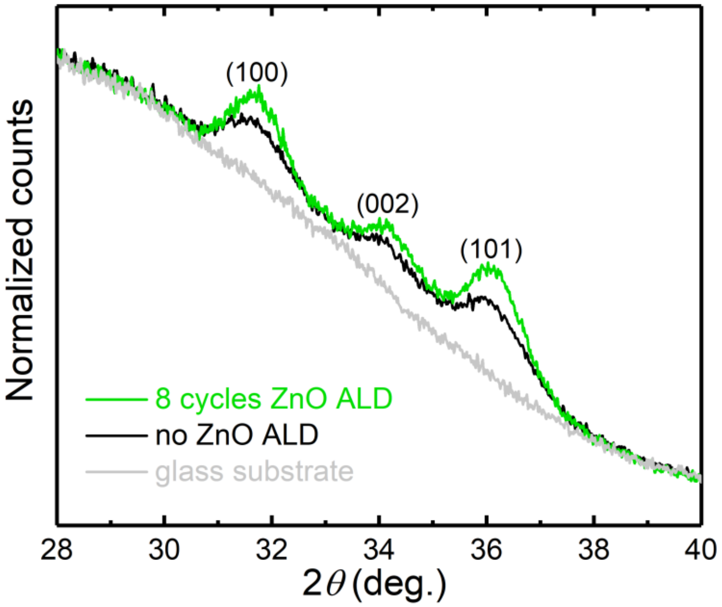

Fig. S5. XRD before and after ZnO ALD. Diffractometry was performed on a single ZnO NC network at two stages of treatment: (1) after sintering IPL (black) and (2) after subsequent ZnO ALD coating (green). To estimate average crystallite size, we subtracted the background signal due to the borosilicate substrate and then fit each peak to a single Gaussian. We obtained the Scherrer broadening by subtracting instrument broadening (determined from a LaB_6 pattern) from each Gaussian's full width at half maximum. In the Scherrer equation we used a shape factor of $0.9 \times 4/3$ to account spheroidal morphology. According to this analysis, ZnO ALD increases the average crystallite size from 9.2 to 9.9 nm. Our description of the ALD coating as "partially epitaxial" is based on the observation that this crystallite size increase is less than twice the ZnO shell thickness estimated from the increase in ZnO volume fraction, $2\delta = 1.2$ nm (see Methods).

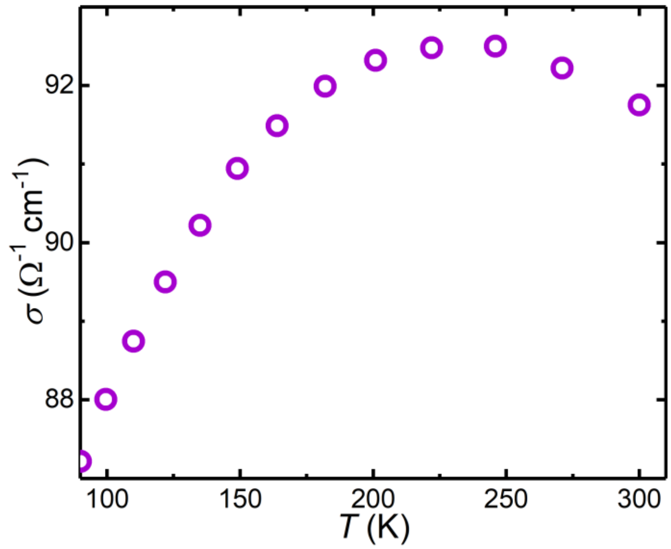

Fig. S6. Rescaled plot of σ(*T*) at $(n\rho^3)_{Hall}$ = 1.5. The data points here are the same as those in Fig. 2a in the text. d*σ*/d*T* clearly changes sign near 250 K.

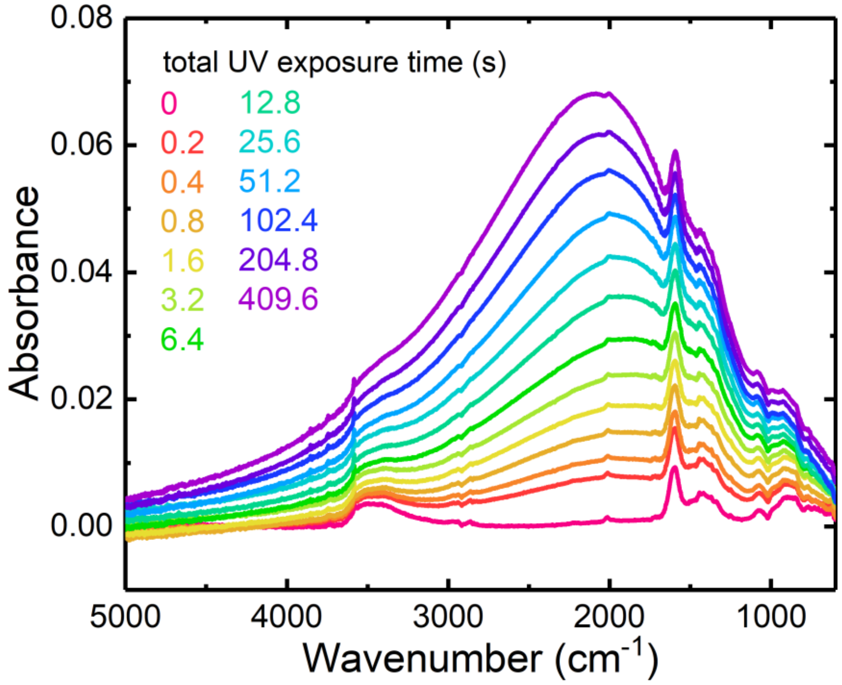

Fig. S7. UV enhancement of LSPR absorption far from the MIT. We obtained a ZnO NC network deep in the insulating regime by eschewing IPL and ALD so that $\rho \approx 0$ and σ(300 K) < 10^{-6} Ω^{-1}cm^{-1}. To modulate the LSPR without inducing sintering, we incrementally irradiated the sample with a relatively low-intensity UV lamp (~50 mW/cm^2 centered at 365 nm) under an N_2 atmosphere (<0.1 ppm O_2 and H_2O). As the total light exposure time was increased from 0.2 to 409.6 s, σ increased by a factor of ~1000 (from ~10^{-6} to 10^{-3} Ω^{-1}cm^{-1}), and the area under the LSPR absorption feature increased by a factor of ~10, while the frequency of peak absorption blueshifted by merely ~20%. Evidently, LSPR blueshift suppression is not unique to samples near the MIT. Also, the large increase in σ (much larger than the increase in LSPR area) is arguably consistent with our surface depletion model, in which there is not only an increase in the total number of free electrons, but also a convergence of the undepleted regions.
